# Characterizing the temporal dynamics of intrinsic brain activities in depressed adolescents with prior suicide attempts

**DOI:** 10.1007/s00787-023-02242-4

**Published:** 2023-06-07

**Authors:** Xiaofang Cheng, Jianshan Chen, Xiaofei Zhang, Ting Wang, Jiaqi sun, Yanling Zhou, Ruilan Yang, Yeyu Xiao, Amei Chen, Ziyi Song, Pinrui Chen, Chanjuan Yang, QiuxiaWu, Taifeng Lin, Yingmei Chen, Liping Cao, Xinhua Wei

**Affiliations:** 1 The Affiliated Brain Hospital of Guangzhou Medical University, 36 Mingxin Road, liwan district, Guangzhou, 510370 Guangdong People’s Republic of China; 2 Key Laboratory of Neurogenetics and Channelopathies of Guangdong Province and the Ministry of Education of China, Guangzhou Medical University, Guangzhou, 510370 Guangdong People’s Republic of China; 3 The Second Affiliated Hospital, School of Medicine, South China University of Technology, 1 Panfu Road, Yuexiu district, Guangzhou, 510180 Guangdong People’s Republic of China; 4 Guangzhou Integrated Traditional Chinese and Western Medicine, Guangzhou, 510800 Guangdong People’s Republic of China

**Keywords:** Major depressive disorder, Suicide, Adolescence, Functional MRI, Frontal cortex

## Abstract

Converging evidence has revealed disturbances in the corticostriatolimic system are associated with suicidal behaviors in adults with major depressive disorder. However, the neurobiological mechanism that confers suicidal vulnerability in depressed adolescents is largely unknown. A total of 86 depressed adolescents with and without prior suicide attempts (SA) and 47 healthy controls underwent resting-state functional imaging (R-fMRI) scans. The dynamic amplitude of low-frequency fluctuations (dALFF) was measured using sliding window approach. We identified SA-related alterations in dALFF variability primarily in the left middle temporal gyrus, inferior frontal gyrus, middle frontal gyrus (MFG), superior frontal gyrus (SFG), right SFG, supplementary motor area (SMA) and insula in depressed adolescents. Notably, dALFF variability in the left MFG and SMA was higher in depressed adolescents with recurrent suicide attempts than in those with a single suicide attempt. Moreover, dALFF variability was capable of generating better diagnostic and prediction models for suicidality than static ALFF. Our findings suggest that alterations in brain dynamics in regions involved in emotional processing, decision-making and response inhibition are associated with an increased risk of suicidal behaviors in depressed adolescents. Furthermore, dALFF variability could serve as a sensitive biomarker for revealing the neurobiological mechanisms underlying suicidal vulnerability.

**Supplementary Information:**

The online version contains supplementary material available at 10.1007/s00787-023-02242-4.

## Introduction

Suicide is among the top leading causes of death in young people aged 15–29 years [1]. Globally, over 150,000 suicide deaths were estimated to have occurred in this age group in 2019 [1]. Suicide attempts are even more common than completed suicides [2]. The ratio of attempted suicides to completed suicides is estimated to be 50:1 to 100:1 among adolescents [3].

Major depression disorder (MDD) is the most common cause of suicide death worldwide [4, 5]. The mortality risk for suicide in young depressed patients is over 20-fold higher than that in the general population [6]. Currently, we have a limited understanding of the neurobiological mechanism underlying suicidal vulnerability in depressed adolescents [7]. In addition, suicide risk assessment still relies on a patient’s voluntary self-reporting and a clinician’s judgment [8] that lacks predictive accuracy. Elucidating the neurological basis of suicidal behaviors in MDD during adolescence would be of great significance for early intervention and treatment, because of the structural and functional brain changes and prominent psychosocial development that occur in this period [9–11], thus facilitating the development of more objective, targeted and effective strategies for preventing suicide.

Functional magnetic resonance imaging (fMRI) studies hold promise for yielding potential markers of suicidal risk by identifying the neurobiological underpinnings of pathophysiologic mechanisms that are not visible at the behavioral level [11]. Thus far, the majority of fMRI studies have focused on adults, with evidence converging on the implication of the corticostriatolimic system that subserves emotion and impulsivity regulation in suicidal behaviors [7, 12–14]; however, functional neuroimaging studies on adolescent suicidal behavior are scarce [7, 11], with Limited task fMRI studies showing abnormal functioning of the salience network, attention network and ventral frontal-limbic system, and resting-state fMRI (R-fMRI) studies identifying altered spontaneous brain activities in the left cerebellum, default-mode network [15] and left prefrontal cortex [16] and decreased internetwork connectivity [17] in juvenile depression with suicide behaviors. However, these conventional functional neuroimaging findings relied on the implicit assumption that brain activity remains stationary during the entire fMRI scan, which may be too simplistic to capture the overall picture because the human brain is a highly dynamic system [18–20].

Brain dynamics, which are characterized by temporal variability and flexibility of brain activities, provide information on the variability in the strength or spatial dynamic organization beyond static perspectives [20–22]. Recent studies have associated dynamic properties of brain activity with the suicidality of MDD [22–24]. Specifically, lower temporal dynamics in brain regions involved in executive and emotional processing have been associated with suicidal ideation in MDD patients [22]. Moreover, combining static and dynamic connectomics could differentiate between depressed patients with and without suicidal ideation [24]. Nevertheless, these studies were all conducted with adults. To our knowledge, previous studies have not investigated the dynamic characteristics of suicidal behaviors in depressed adolescents. Given the immaturity of the involved brain systems in adolescents [7], it is necessary to explore the unique characteristics of the brain dynamics in this age group.

Local brain activities are reflections of the intrinsic brain fluctuations generated from mental and cognitive processes [25–27]. Combining an effective metric, namely, the amplitude of low-frequency fluctuation (ALFF) [28], a sliding-window approach can be used to measure time-varying brain activities (dynamic ALFF, dALFF) as the variance of ALFF over time (dALFF variability). The current study used the dALFF method to investigate the neurobiological mechanism that confers suicidal vulnerability in depressed adolescents. Based on the aforementioned studies, we sought to test the following hypotheses: (1) The time-varying pattern of brain activities may be altered in depressed adolescents with suicide attempts; and (2) Altered dALFF variability is correlated with clinical variables and can constitute a potential neuromarker for classifying depressed adolescents with and without suicide attempts as well as for predicting the severity of suicidal ideation. In addition, we also performed an exploratory analysis to identify the neural substrate differences between MDD with single SA (MDD-SSA) and MDD with recurrent SA (MDD-RSA).

## Materials and methods

### Participants

The current study included 86 treatment-seeking depressed adolescents recruited from the Affiliated Brain Hospital of Guangzhou Medical University and 47 healthy controls (HCs) recruited through community postings. All participants were of Han Chinese ethnicity and right handed, and the age range was from 10 to 17 years.

Depressed adolescents were eligible if they (1) met a diagnosis of first-episode major depressive disorder (MDD) based on DSM-V criteria and (2) were drug-naive or consecutively used psychotropic medication for less than 14 days [29]. Healthy adolescents were eligible if they had no past or current psychiatric diagnoses, and they were matched with the MDD group in terms of age and education. The exclusion criteria for both groups included psychoactive substance abuse, neurological illness, a history of loss of consciousness (≥ 5 min) caused by brain injury, severe general physical illness, MRI scanning contraindications, and IQ lower than 70 based on the Wechsler Abbreviated Scale of Intelligence. Healthy adolescents with suicide behaviors or a family history of any psychiatric disorder and MDD patients with developmental disorders (except attention deficit and hyperactivity disorder) were also excluded. Ten adolescents with MDD and two healthy adolescents were excluded for excessive head movements and poor image quality, and the remaining 76 depressed adolescents and 45 HCs were included in the final analyses. The 76 eligible depressed adolescents were further divided into the MDD-SA group (38 individuals with at least one suicide attempt) and the MDD-NSA group (38 individuals without prior suicide attempts). Notably, given that previous studies indicated that the clinical characteristics of single and recurrent attempters were different [30–32], we stratified the MDD-SA group into two subgroups, namely, the MDD-SSA (*N* = 15) and MDD-RSA (*N* = 19), to explore the underlying neurobiological mechanism differences between them (the total number of suicide attempts of four participants was missing in our dataset).

Written informed consent and assent were obtained from all participants and their parents prior to participation. The current study was part of the work of a multidimensional cohort study on the Symptomatic trajectory and Biomarkers of Early Adolescent Depression (sBEAD), of which the study protocol has been published before (Chinese Clinical Trial Registry Identifier: ChiCTR2100049066) [33]. The institutional review board of Affiliated Brain Hospital of Guangzhou Medical University approved the study protocol, and this study was conducted in accordance with the amended principles of the Declaration of Helsinki.

### Clinical assessment

All participants underwent clinical evaluations using the Schedule for Affective Disorders and Schizophrenia for School-Aged Children (K-SADS-PL) matched for DSM-V by qualified psychiatrists to screen for diagnosis.

The lifetime history of suicidal behaviors was assessed using the Columbia Suicide Severity Rating Scale (C-SSRS). The C-SSRS has demonstrated good convergent, divergent, predictive validity in adolescent samples [34, 35]. We assessed suicidal ideation (SI) severity for each participant once the SI severity was endorsed. We focused on the baseline C-SSRS lifetime SI intensity score because it is a significant predictor of subsequent suicide attempts during the 6 month follow-up period [36]. The SI intensity score was derived from five rating items: frequency, duration, controllability, deterrents and reasons for ideation [36, 37]. Suicide attempts were defined as self-injurious behaviors with any intention to die [38, 39]. It should be noted that suicide attempt in our study was referred to an actual suicide attempt, ambiguous attempts such as interrupted, aborted suicide attempt, preparatory acts toward attempt, or non-suicidal self-harm were not included in SA group. The number and methods of suicide attempts, medical lethality and trigger events were also recorded. The severity of depression and anxiety was evaluated with the 24-item Hamilton Depression Scale (HAMD) and Hamilton Anxiety Scale (HAMA).

### MRI acquisition

All participants were scanned during the eyes-closed rest condition on a 3.0 Tesla MRI system (Achieva X-series, Philips Medical Systems, Best, Netherlands) in the Department of Radiology at The Affiliated Brain Hospital of Guangzhou Medical University. Foam pads and headphones were used to minimize head motion and reduce scanner noise. Participants were not allowed to take medicine or drink stimulating beverages before the day of scanning. None of the subjects fell asleep during the scan, which was confirmed by a questionnaire following the scan. R-fMRI data were acquired using a gradient-echo echo-planar imaging sequence with the following parameters: repetition time (TR) = 2000 ms, TE = 30 ms, flip angle (FA) = 90°, field of view (FOV) = 220 × 220 mm^2^, matrix = 64 × 64, axial slices = 33, thickness = 4 mm, gap = 0.6 mm, and voxel size = 3.44 × 3.44 × 4 mm^3^. The scan lasted for 8 min 12 s (240 volumes). Three-dimensional T_1_-weighted images were obtained using a fast gradient echo sequence with the following parameters: TR/TE = 8.2 ms/3.8 ms, FOV = 256 × 256 mm^2^, matrix = 256 × 256, sagittal slices = 188, thickness = 1 mm, and voxel size = 1 × 1 × 1 mm^3^. Axial *T*_2_-weighted images were also collected to exclude anatomical abnormalities.

All functional imaging data preprocessing was performed using the Data Processing and Analysis for Brain Imaging (DPABI V4.3, http://rfmri.org/dpabi) and Statistical Parametric Mapping (SPM12, http://www.fil.ion.ucl.ac.uk/spm) software packages. Preprocessing steps included discarding the first 10 volume images and performing slice timing correction, head motion correction, normalization of the functional images to the Montreal Neurological Institute (MNI) space using unified segmentation of the *T*_1_-weighted images with 3 × 3 × 3 mm^3^ resampling, spatial smoothing with a 4 mm Gaussian kernel, linear detrending, and regression of confounding covariates (Friston-24 motion parameters, white matter, cerebrospinal fluid). As previous studies reported that global signal regression can introduce spurious anti-correlations and substantially alter interregional correlations, we did not remove global signal in the preprocessing procedure [40, 41]. The mean framewise displacement (FD) [42] was calculated to evaluate the head movement of each participant. Subjects were excluded under a head motion criterion of 2.5 mm translation and 2.5° rotation as well as a mean FD of 0.25 mm.

### Dynamic ALFF computing

Dynamic ALFF was measured with a sliding-window approach using temporal dynamic analysis (TDA) toolkits based on DPABI. Window size is a controversial parameter for dynamic analysis. Previous studies revealed the window size in the range of 30 s to 1 min was suitable for capturing brain dynamics [43–45]; however, another study held different opinions that that the minimum window length should be no less than 1/*f*_min_ (1/0.01 = 100 s) [46]. The present study chose 30 TRs (60 s) as window size and we also performed auxiliary analyses with window sizes of 50 TRs (100 s) and 40 TRs (80 s), respectively (Results are provided in supplementary materials). Hamming window was applied to shift the BOLD signals with a window step of 1 TR (2 s). Based on this, the full-length time series of each subject were divided into 201 windows, and the ALFF maps were calculated within each window at the frequency range of 0.01–0.08 Hz, thus generating a set of ALFF maps for each individual. To evaluate the temporal variability (dALFF variability), we then computed the standard deviation (SD) of these dALFF maps across windows. Finally, for standard purposes, the dALFF variability of each voxel was further transformed into z-scores by subtracting the mean and dividing by the SD of global values within a grey mask. To verify whether dALFF and static ALFF (sALFF) shared similar patterns or provided complementary information for uncovering the neurobiology conferring suicidality to depressed adolescents, we examined the sALFF map for each subject as well.

### Statistical analysis

#### Demographic and clinical characteristics

Differences in demographic and clinical characteristics were assessed using SPSS 22.0 software (SPSS Inc.; Chicago, IL, USA). One-way analysis of variance, *χ*^2^ tests, two-sample *t*-tests or Mann–Whitney *U* test were utilized when appropriate, and significance was set to *P* < 0.05.

#### Between-group differences of dALFF variability

The maps of dALFF variability were averaged across all the participants to explore the distribution patterns of dynamic local brain activities within each group. A one-way ANOVA was performed to compare the group differences in dALFF variability among the MDD-SA, MDD-NSA and HC groups, with age, sex, education level and mean FD as covariates. Post hoc analyses were also implemented to assess the dALFF variability differences between each pair of groups within a mask that included all the significant clusters from the ANOVA. Multiple comparisons were performed using Gaussian random field (GRF) theory (voxel-level *P* < 0.01, cluster-level *P* < 0.05, one tail for ANOVA and two-tailed for two-sample *t*-tests). Finally, a two-sample *t*-test was run again to evaluate the dALFF variability differences between the MDD-RSA and MDD-SSA groups within clusters with significant differences between the MDD-SA group and the other two groups.

#### Correlation analysis

A partial correlation analysis was conducted to explore the relationship between mean dALFF variability within clusters with significant differences between the MDD-SA and MDD-NSA groups and clinical variables after controlling for confounders, including age, sex, education level and mean FD. The significance level was set to a Bonferroni-corrected *P* < 0.05/12.

#### Classification analysis based on dALFF variability

We combined Logistic regression and receiver operating characteristic (ROC) curve analyses, to test the classification performance of dALFF variability in distinguishing MDD-SA from MDD-NSA. We chose mean dALFF attributes that had significant correlations with the SI intensity scores and mean dALFF attributes of all significant clusters as classification features, respectively. Similar classification analyses were also run for sALFF. Finally, we combined all the mean dALFF and sALFF attributes of significant clusters to test whether they can improve the classification power.

#### Predicting SI intensity based on dALFF variability

To further investigate the relationship between dALFF variability and SI symptoms measured by the C-SSRS. We predicted SI intensity for each individual in the whole patient group using a general linear model. We chose a leave-one-out cross-validation (LOOCV) scheme to it has been demonstrated to be able to yield a robust and reliable model [22, 47]. Specifically, data from a single participant were left out as a test dataset, and the remaining subjects’ data were used as a training set (using the same feature selection strategies as that in the classification analysis) to build a prediction model, based on which the SI intensity scores of the testing dataset were predicted. Finally, Pearson’s correlation analysis was performed to determine whether the predicted SI intensity scores were correlated with the observed SI intensity scores in the MDD group. If a significance level of *P* < 0.05 was established, we considered that these features could predict SI intensity and vice versa.

#### Reproducibility validation

We validated our findings of dALFF variability by considering several potential confounding factors. First, to evaluate the influence of sliding window sizes on our results, we conducted auxiliary analyses by recalculating the main results using two other sliding window lengths (40 and 50 TRs). Secondly, as age at onset and illness duration differed between the MDD-SA and MDD-NSA groups, we reperformed a post hoc analysis between these two groups with age at onset and illness duration as additional covariates, respectively.

## Results

### Demographic and clinical characteristics

As displayed in Table 1, no significant differences in age (*F* = 1.19, *P* = 0.308), education level (*F* = 1.96, *P* = 0.146) were observed among the three groups, although significant difference were observed in gender (*χ*^2^ = 8.91, *P* = 0.012). Significant differences were not observed in medication counts (*χ*^2^ = 0.48, *P* = 0.923) or in HAMA (*T* = 1.89, *P* = 0.063) between the MDD-SA and MDD-NSA groups. In addition, the MDD-SA group scored significantly higher in HAMD (*T* = 2.41, *P* = 0.018) and SI intensity (*T* = 4.29, *P* < 0.001) than the MDD-NSA group. The age at onset (*Z* = − 2.88, *P* = 0.004) of MDD-SA group was earlier than that of the MDD-NSA group, and the illness duration of MDD-SA group was longer than that in MDD-NSA group (*Z* = − 2.27, *P* = 0.024). We also observed significant differences of FD values between MDD-SA and MDD-NSA groups (*Z* = − 2.08, *P* = 0.040).

**Table 1:** Demographic and clinical features of study participants (Mean ± standard deviation (SD))

Variables	SA (*n* = 38)	NSA (*n* = 38)	HC (*n* = 45)	Statistics (*P*) *F*,* t, Z*, *χ*2
Gender (M/F)	6/32	13/25	21/24	*χ*^2^ = 8.91 (0.012)^*^
Age	14.1 ± 1.5	14.6 ± 1.6	14.0 ± 2.3	*F* = 1.19 (0.308)
Education	8.2 ± 1.7	8.9 ± 2.0	8.0 ± 2.5	*F* = 1.96 (0.146)
HAMD	28.5 ± 8.6	23.8 ± 8.3	–	*t* = 2.41 (0.018)^*^
HAMA^a^	19.6 ± 7.3 (*n* = 35)	16.4 ± 6.8 (*n* = 32)	–	*t* = 1.89 (0.063)
SI intensity^a^	15.4 ± 2.7 (*n* = 38)	11.0 ± 5.6 (*n* = 36)	–	*t* = 4.29 (< 0.001)^*^
Age of illness onset^a^	12.9 ± 1.6 (*n* = 38)	14.1 ± 1.8 (*n* = 37)	–	*Z* = − 2.88 (0.004)^*^
Illness duration, months	16.7 ± 14.0	11.2 ± 10.2	–	*Z* = − 2.27 (0.024)^*^
Weight^a^	53.3 ± 14.1 (*n* = 31)	54.0 ± 12.9 (*n* = 28)	–	*Z* = − 1.164 (0.245)
FD	0.75 ± 0.27	0.64 ± 0.26		*Z* = − 2.08 (0.040)^*^
Unmedicated/medicated^a^	16/20	13/25	–	*χ*^2^ = 0.81 (0.254)
Medication use (*n*)				
Antipsychotics^a^	20.7% (6/29)	7.14% (2/28)		*χ*^2^ = 0.48 (0.923)
Antidepressants^a^	48.3% (14/29)	28.6% (8/28)		
Mood stabilizer^a^	10.3% (3/29)	3.6% (1/28)		
Benzodiazepines^a^	24.1% (7/29)	10.7% (3/28)		

*SA* prior suicide attempt, *NSA* no prior suicide attempt, *HC* healthy control, *M* male, *F* female, *HAMD* Hamilton Rating Scale for Depression, *HAMA* Hamilton anxiety Scale;

^a^Information was missing for some participants; *SI* suicide ideation, *χ*^2^*,* statistic of Chi-square test; *F* statistic of one-way analysis of variance, *Z* statistic of Mann–Whitney *U* test, *t*, statistic of two-sample *t*-test; *FD* framewise displacement

^*^P < 0.05

### Spatial distributing patterns of dALFF variability

MDD-SA, MDD-NSA, and HC displayed similar spatial distribution patterns of dALFF variability, which was nonuniform across the brain. We then performed Pearson's correlation analysis to evaluate the relationship between sALFF and dALFF variability within the MDD-SA, MDD-NSA, and HC groups. The results showed that sALFF metrics were all highly and positively correlated with dALFF variability in these three groups (Figure S2). Brain regions with high temporal variability were mainly distributed in the occipital cortices and postmedial, lateral frontal, parietal, limbic and sensorimotor cortices. Brain regions with low variability located in the inferior temporal and medial prefrontal cortices (Figure S1). The spatial distribution pattern and between-group differences of sALFF maps were highly consistent with that of the dALFF variability (Figure S3).

### Between-group differences of dALFF variability

**Fig. 1:**
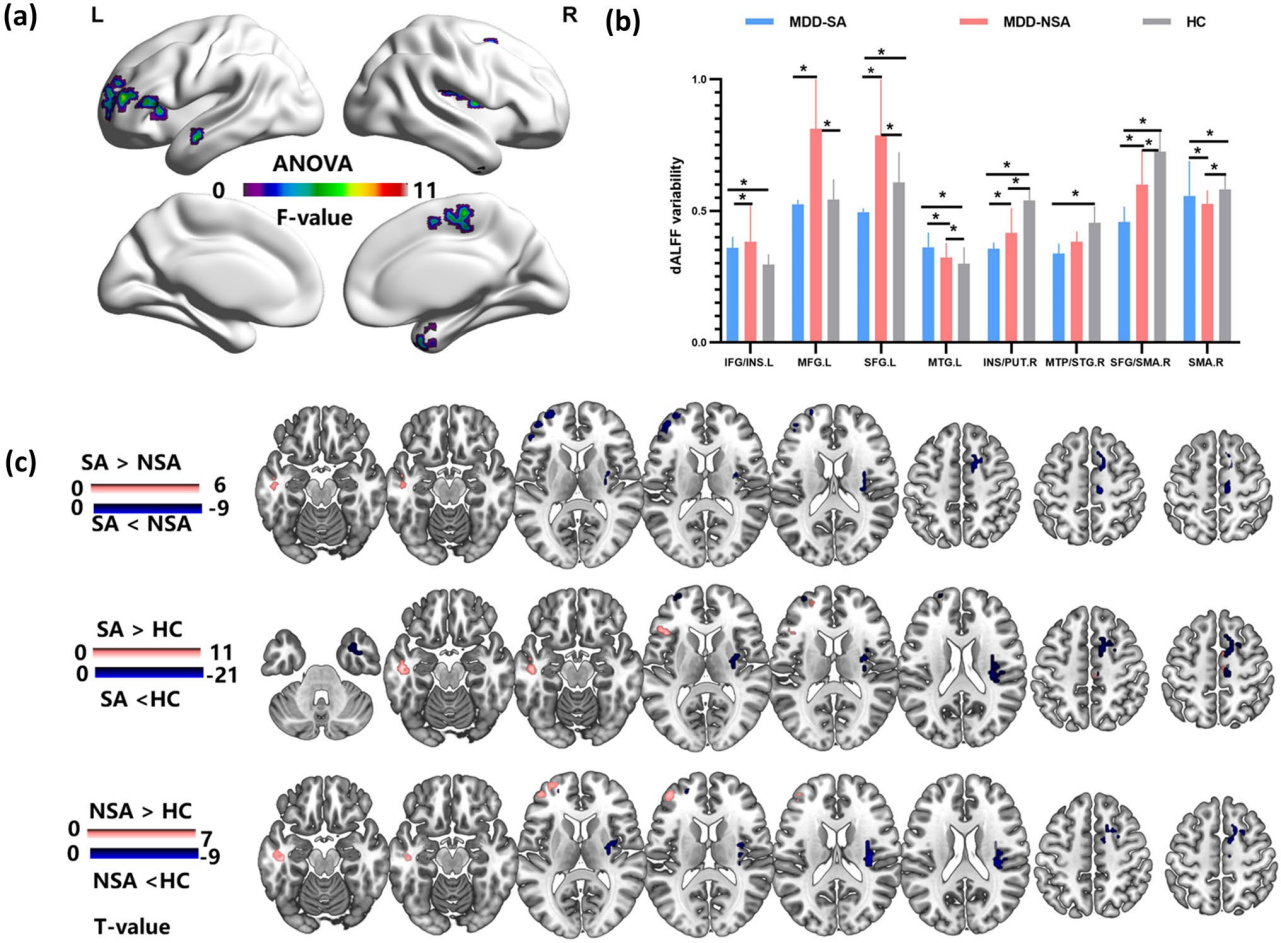
**a** ANOVA results showing dALFF variability differences across three groups. **b**–**c** Post hoc results among the MDD-SA, MDD-NSA, and HC groups. *IFG* inferior frontal gyrus, *INS* insula, *MFG* middle frontal gyrus, *SFG* superior frontal gyrus, *MTG* middle temporal gyrus, *PUT* putamen, *MTP* middle temporal pole, *STG*, superior temporal gyrus, *SMA* supplementary motor area, *L* left, *R* right. *dALFF* dynamic amplitude of low-frequency fluctuations, *MDD* major depressive disorder, *SA* prior suicide attempt, *NSA* no prior suicide attempt, *HC* healthy control

The ANOVA results showed significant dALFF variability differences across the three groups spread over eight clusters, including the right middle temporal pole (MTP)/superior temporal gyrus (STG), left middle temporal gyrus (MTG), left inferior frontal gyrus (IFG) extending to the left insula, left middle frontal gyrus (MFG), left superior frontal gyrus (SFG), right SFG, right supplementary motor area (SMA), and right insula extending to the right putamen (Fig. 1a).

The post hoc analysis revealed that both the MDD-SA and MDD-NSA groups showed elevated dALFF variability in the left MTG and reduced dALFF variability in the right insula, putamen, SMA and SFG relative to the HC group. In addition, the MDD-SA group exhibited higher dALFF variability in the left MTG and lower dALFF variability in the left IFG, MFG, SFG, right SMA, SMA/SFG and right insula extending to the putamen than the MDD-NSA group (Fig. 1b–c, Table 2).

**Table 2:** Brain regions showing between-group differences of dALFF variability

Brain regions	BA	Volume (mm^3^)	MNI coordinate			*T* values
			*x*	*y*	*z*	
SA > NSA						
Left MTG	20	432	− 51	− 18	− 18	5.2992
SA < NSA						
Left IFG	45	216	− 48	36	12	− 5.1366
Left MFG	46	702	− 42	48	12	− 5.6684
Left SFG	10	729	− 30	60	9	− 5.5237
Right INS/PUT	48	648	− 33	− 6	15	− 5.4750
Right SFG /SMA	6/4	675	15	6	51	− 7.3319
SA > HC						
Left MTG	20	648	− 48	− 18	− 15	11.3506
Left IFG	45	513	− 36	21	6	10.3684
SA < HC						
Left MFG	46	513	− 30	60	9	− 4.1496
Left SFG	10	324	− 24	57	27	− 4.6257
Right INS/PUT	48	1404	33	− 6	15	− 20.6512
Right SFG/SMA	6	837	15	9	51	− 12.5465
Right SMA	4	486	9	− 24	60	13.1642
Right MTP/ITG	20	432	39	0	− 36	− 9.5281
NSA > HC						
Left MTG	20	432	− 48	− 21	− 18	6.3175
Left MFG	46	675	− 42	48	12	5.6956
NSA < HC						
Right SMA/SFG	6/8	487	6	− 21	60	− 6.2548
Right INS/PUT	48	1920	30	− 9	6	− 9.0777
Right SMA	4	486	6	− 21	60	− 6.2548

*BA* Brodmann’s area, *MNI* Montreal neurological institute; *MTG* middle temporal gyrus, *IFG* inferior frontal gyrus, *MFG* middle frontal gyrus, *SFG* superior frontal gyrus, *INS* insula, *PUT* putamen, *SMA* supplementary motor area, *MTP* middle temporal pole, *ITG* inferior temporal gyrus, *dALFF* dynamic amplitude of low-frequency fluctuations

**Fig. 2:**
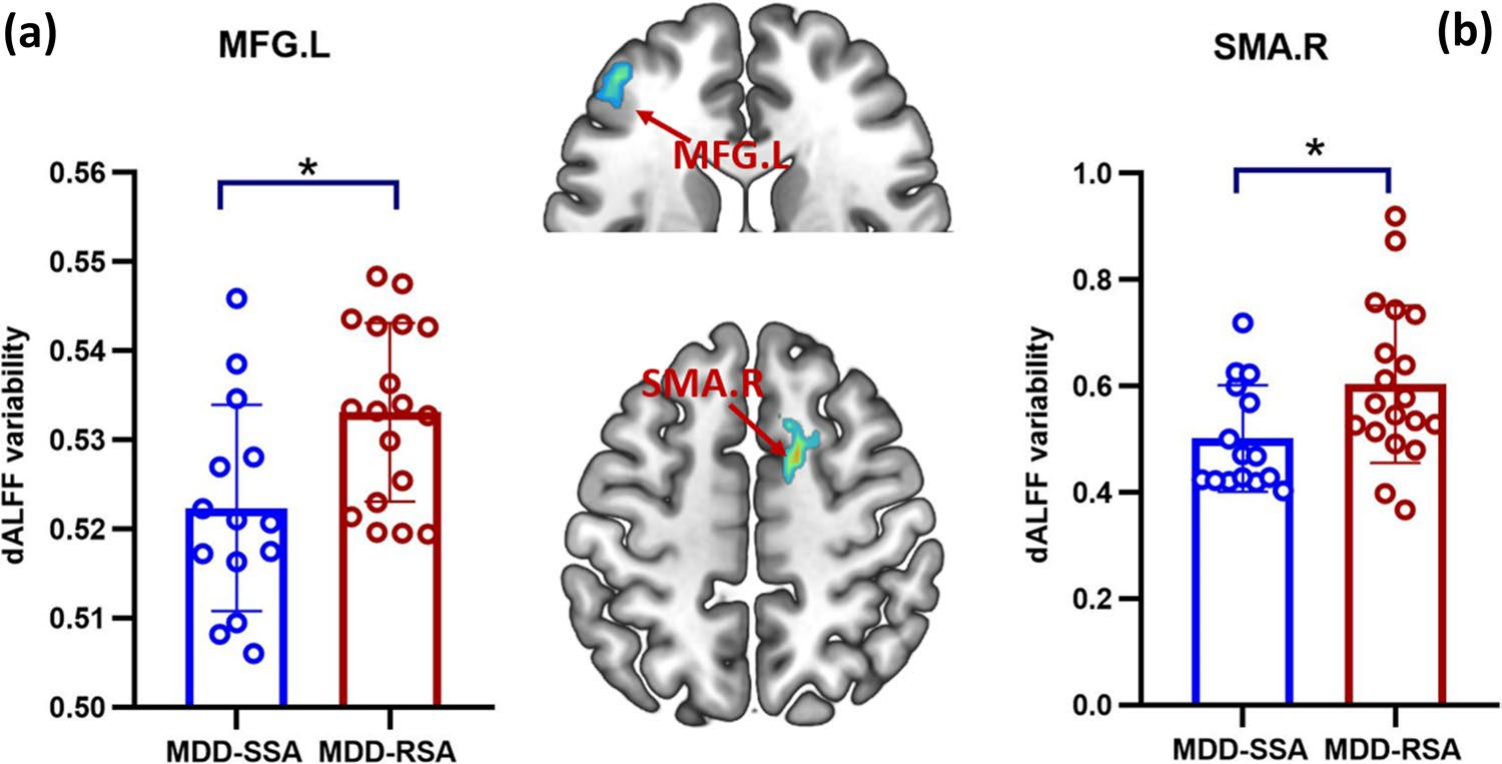
dALFF variability difference between MDD-SSA and MDD-RSA in the **a** left middle frontal gyrus and **b** right supplementary motor area. * Uncorrected *P* < 0.05. *dALFF* dynamic amplitude of low-frequency fluctuations, *MFG* middle frontal gyrus, *SMA* supplementary motor area, *L* left, *R* right, *MDD *major depressive disorder, *SSA* single suicide attempt, *RSA* recurrent suicide attempts

Two-sample t-test revealed that the dALFF variability was higher in the left MFG (*T* = 2.36, uncorrected *P* = 0.026) and right SMA (*T* = 2.30, uncorrected *P* = 0.028) in the MDD-RSA group than in the MDD-SSA group; however, no cluster survived correction for multiple comparisons (Fig. 2).

### Correlation analysis

**Fig. 3:**
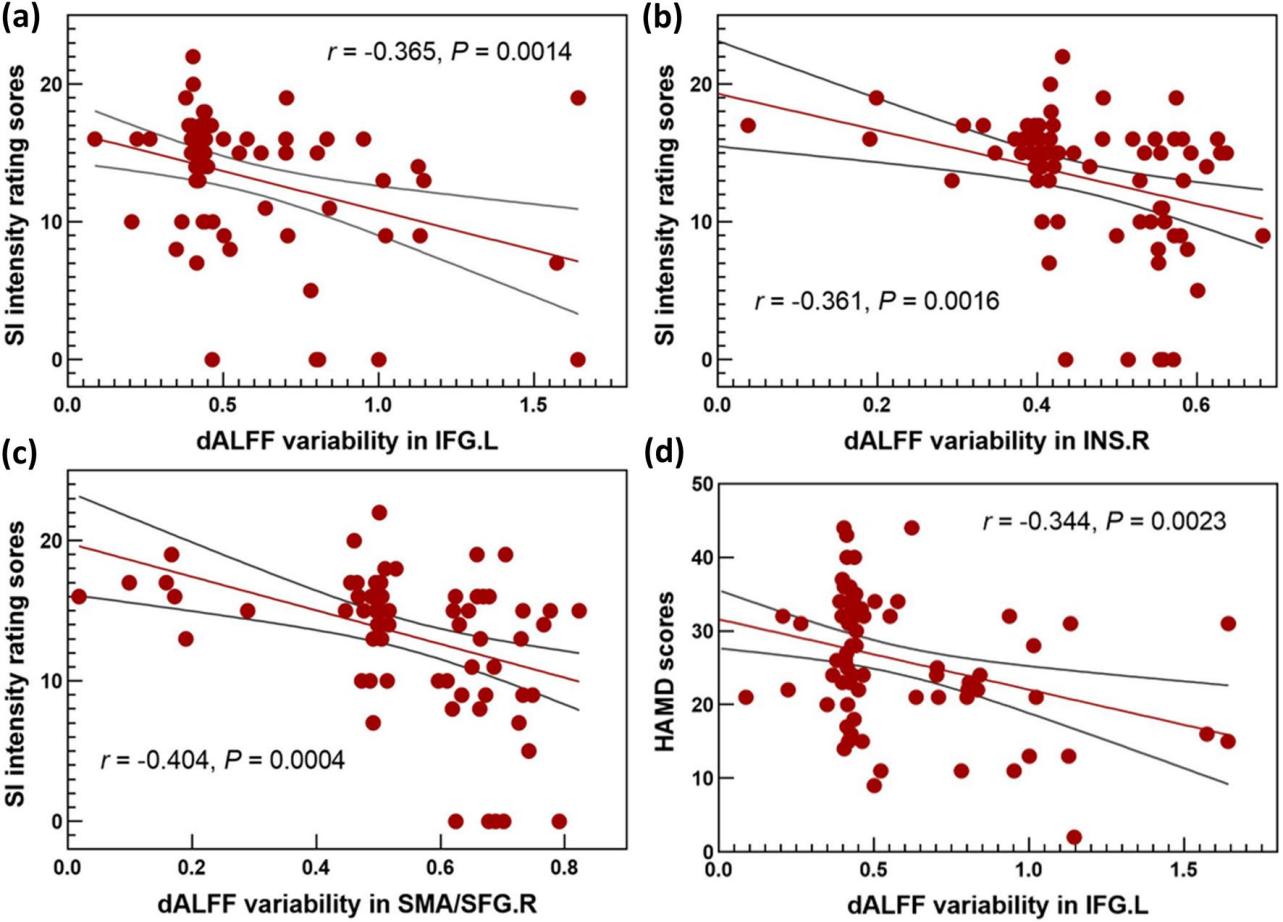
Correlation between dALFF variability and clinical variables. **a**–**c** dALFF values of the left IFG, right insula and right SMA/SFG were negatively correlated with suicide ideation intensity scores in the whole patient group; **d** dALFF values of the left IFG were negatively correlated with depression severity evaluated by HAMD in the whole patient group; The shadow indicates 95% confidence intervals. *IFG* inferior frontal gyrus; *INS* insula, *SMA* supplementary motor area, *SFG* superior frontal gyrus; *L* left; *R* right; *HAMD* Hamilton depression rating Scale; *SI* suicide ideation; *dALFF* dynamic amplitude of low-frequency fluctuations

The correlation analysis showed significantly negative correlations between SI intensity scores and dALFF variability values in the left IFG (*r* = − 0.365, *P* = 0.0014), right insula (*r* = − 0.361, *P* = 0.0016) and SMA/SFG (*r* = − 0.404, *P* = 0.0004) in the whole patient group (Fig. 3a–c). The dALFF variability values in the left IFG were negatively correlated with HAMD scores (*r* = − 0.344, *P* = 0.0023) (Fig. 3d). No significant correlation between dALFF variability and clinical variables was found within MDD-SA or MDD-NSA group.

### Classification analysis based on dALFF variability

**Fig. 4:**
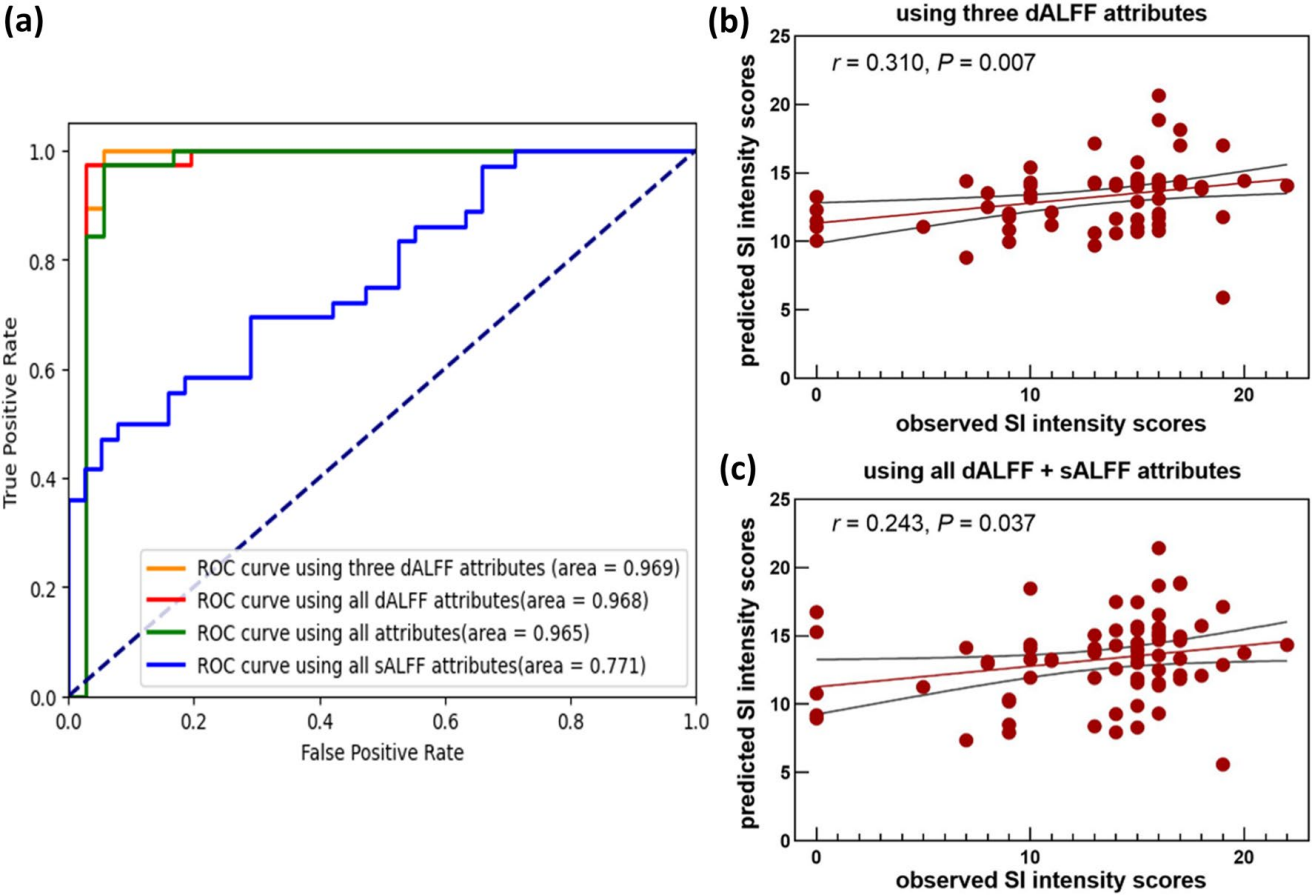
Classification and prediction based on dALFF variability and sALFF. **a** Differentiating MDD-SA from MDD-NSA by selecting different features; (**b**–**c**) Predicting SI intensity scores by combining dALFF variability values in the left inferior frontal gyrus, right insula and supplementary motor area/superior frontal gyrus; **b** and dALFF and sALFF attributes of all significant clusters between the MDD-SA and MDD-NSA groups **c**. The shadow indicates 95% confidence intervals. dALFF, dynamic amplitude of low-frequency fluctuations; *sALFF* static amplitude of low-frequency fluctuations; *SI* suicide ideation

The classification analysis showed that combining the dALFF variability values in the left IFG, right insula and SMA/SFG had the best diagnostic performance (area under the curve (AUC): 0.969, 95% confidence intervals (CI) 0.902–1; sensitivity: 94.44%; specificity: 100%) in discriminating MDD-SA from MDD-NSA, followed by combining the dALFF attributes of all significant clusters (AUC: 0.968, 95% CI 0.900–1; sensitivity: 86.11%; specificity: 97.37%), combining the dALFF and sALFF attributes of all significant clusters (AUC: 0.965, 95% CI 0.900–1; sensitivity: 83.33%; specificity: 100%) and using the sALFF attributes of all significant clusters (AUC: 0.771, 95% CI 0.660–0.869; sensitivity: 80.55%; specificity: 47.36%) (Fig. 4a). Overall, the discriminating power of dALFF variability in differentiating MDD-SA from MDD-NSA was higher than that of sALFF.

### Predicting SI intensity based on dALFF variability

Similar to the classification results, combining the dALFF variability values in the left IFG, right insula and SMA/SFG (*r* = 0.310, *P* = 0.007) exhibited the best power in predicting SI intensity, followed by using the dALFF and sALFF attributes of all significant clusters between the MDD-SA and MDD-NSA groups (*r* = 0.243, *P* = 0.036); however, using the dALFF attributes (*r* = 0.138, *P* = 0.241) and sALFF attributes (*r* = 0.061, *P* = 0.607) of all significant clusters could not predict SI intensity (Fig. 4b–c).

### Reproducibility validation

Overall, the SA-related alterations in dALFF variability in the different validation strategies were highly consistent with our main results, suggesting a high reproducibility of the results (Figure S3-S7).

## Discussion

By using a novel method of exploring brain dynamics, we first identified SA-related alterations in dALFF variability primarily in the left MTG, IFG, MFG, SFG, right SFG, SMA and insula in depressed adolescents. Notably, the altered dALFF variability could be used to generate diagnostic models in discriminating MDD-SA from MDD-NSA with high accuracy, and some features could predict the severity of SI. Moreover, exploratory comparisons also revealed some differences in dALFF variability in the left MFG and right SMA between the MDD-RSA and MDD-SSA groups. Together, these findings provide new insights into the neurobiological mechanisms underlying suicidality in depressed adolescents.

Brain dynamics reflect the functional capacity of the neural system and could serve as a novel neuromarker for various neuropsychiatric disorders [48–50], including MDD [22, 51]. Currently, abnormal dynamics of local brain activity [22] and interregional functional connectivity [23, 24] have been demonstrated to be associated with suicidality in adults with MDD. Expanding on previous studies on adults, the current work identified SA-related alterations in dALFF variability in depressed adolescents for the first time.

The IFG (BA 45) and SFG (BA 10), as parts of the ventrolateral prefrontal cortex (VLPFC), play an important role in cognitive control and response inhibition [7], and these behaviors are usually impaired in suicide attempters [52]. The MFG (BA 46) is an integral component of the dorsolateral prefrontal cortex (DLPFC) involved in decision-making over value processing, which is another higher-order cognitive function implicated in suicide diathesis [7, 52, 53]. High IFG and SFG activation have been associated with past suicidal behaviors in psychotic major mood disorders during cognitive control task performance [54]. Similarly, an R-fMRI study found significantly decreased zALFF values in the left SFG and MFG in young depressed patients with suicide behavior [16]. Recent studies have also reported blunted activation of the DLPFC in response to value differences in patients with suicide attempts [55], particularly in well-planned SAs [56]. The SMA (medial portion of BA 6) is one of the key regions that are functionally linked to form the cognitive control loop [57, 58]. Converging neurophysiological and imaging findings indicate that SMA is crucial for correct response inhibition [59, 60]. The functional connectivity between the right SMA and medial frontal cortex had been shown to be significantly correlated with SI intensity in depression [61] as well as past suicide behavior in schizophrenia [57]. Consistent with the limited suicide studies on adolescents, we found lower brain dynamics primarily located in the VLPFC, DLPFC and SMA, which are associated with cognitive control, decision-making and response inhibition. As indicated by previous studies[62, 63], a dynamic brain is important for cognitive functioning while a less dynamic brain activity is related to worse performance on cognitive tasks. Taken together, we speculated that reduced dALFF variability in the VLPFC, DLPFC and SMA may contribute to diminished top-down inhibitory control of behavior and impaired decision-making and planning[7, 64], thus leading to an increased risk of suicide attempts in juvenile depression.

The insula cortex is a key hub of the salience network that mediates or switches between the extended ventromedial prefrontal cortex and dorsal prefrontal cortex/IFG system [65, 66], which possibly facilitates the transition from SI to attempt [7]. Lower insula volume [67, 68] and cortical thickness [69] have been demonstrated in adult SAs across different psychiatric disorders, and altered insula volume was related to attempted lethality and impulsivity [67, 70]. Besides, the disrupted functional connectivity between the insula and SMA was reported to be closely associated with SA in young depressed patients [71]. Furthermore, our previous study showed the functional connectivity strength in the right insula was decreased in patients with bipolar disorder and SA [72]. The underlying biological mechanism that insula affecting suicidal behaviors is still unclear. previous studies indicated that immune challenges activated interoceptive brain pathways (including the insula), triggering alterations in the emotion, motivation and cognition [73]. Additionally, one positron emission tomography study reported that patients with MDD and SI had increased translocator protein activity than those without SI, this result was consistent with postmortem findings of increasing microglia cell activation in MDD with SI, suggesting that the insula may mediate suicidal thoughts and behaviors through neuroinflammation pathway [74]. In line with these findings, the current study suggests that the insula may also underlie the neural mechanism of suicidal behaviors in depressed adolescents.

The temporal association cortices are associated with the perception of intentional behavior and retrieval of personal experiences from memory, and they are also involved in semantic and emotional processing [16, 75]. Consistently, both decreased volume [76] and zALFF values [16] in the MTG were observed in young depressed patients with SA, and the intrinsic functional connectivity strength between the MTG and right anterior cingulate cortex was positively correlated with SI. When viewing 50% intensity angry faces, adolescents with history of suicide attempt and depression showed significantly greater activity in right middle temporal cortex than adolescents with history of depression alone. Additionally, Parkar et al. found individuals with suicidal behaviors had decreased cerebral glucose metabolism in temporal cortex [77]. Consistent with these studies, our study demonstrated elevated dALFF variability in the left MTG, which may underlie emotional dysregulation in youth with depression and suicidal behaviors.

Previous studies indicated that the clinical characteristics of MDD-SSA and MDD-RSA were different [30–32, 78], nevertheless, neuroimaging studies have not been performed to investigate the underlying neurobiological mechanism between them. In our exploratory comparison, we found some differences in dALFF variability in the left MFG and right SMA between MDD-RSA and MDD-SSA. However, these two clusters cannot survive multiple comparison correction, which may be related to the small sample size. Given their important roles in decision-making and inhibition control mentioned before, we postulated that the aggravation of disturbance in the left MFG and right SMA may cause impulsive decision-making and incorrect response inhibition, thus leading to an increasing risk of recurrence of suicide attempts.

## Limitations

Several limitations of the current study warrant consideration. First, although we believe that our fMRI data could provide adequate information for performing a dynamic analysis, it should be noted that fMRI scans with higher temporal resolution (i.e., multiband technique) would be better for investigating the temporal dynamics of intrinsic brain activities. Second, the LOOCV model used in predicting SI intensity is an unbiased and suitable method for analyzing datasets with small sample sizes; however, it may produce higher variance in prediction errors [79]. Third, although depressed participants received consecutive psychotropic medications for less than two weeks, we could still not completely rule out the possible influence of medications on our results. Fourth, the current study was cross-sectional, and studies with longitudinal designs are essential for identifying the risk markers of future suicide attempts. Last, due to the small size, the results should be interpreted with caution and require replication in further research with a larger sample size.

## Conclusions

In summary, the current study identified SA-related alterations in dALFF variability primarily in the left MTG, IFG, MFG, SFG, right SFG, SMA and insula in depressed adolescents. Disturbances of the brain dynamics in these brain regions may contribute to emotional dysregulation, impaired decision-making and incorrect inhibition control, thus leading to an increased risk of suicidal behaviors. Moreover, dALFF variability was capable of generating better diagnostic and prediction models for suicidality than static ALFF. Our findings provide new insights into the neurobiological mechanisms underlying suicidal behaviors in youth with depression.

### Supplementary Information

Below is the link to the electronic supplementary material.

Supplementary file1 (DOCX 4729 KB)

Supplementary file2 (DOCX 1975 KB)

## Data Availability

The data that support the findings of this study are available from the corresponding author.
