# Supplementary material for: Characterizing the temporal dynamics of intrinsic brain activities in depressed adolescents with prior suicide attempts
Source: Eur Child Adolesc Psychiatry. 2023 Jun 7;33(4):1179–91. doi: 10.1007/s00787-023-02242-4 (PMC11032277; doi:10.1007/s00787-023-02242-4)
Supplement: Supplementary file 1 — Supplementary file1 (DOCX 4729 KB) [file 787_2023_2242_MOESM1_ESM.docx]

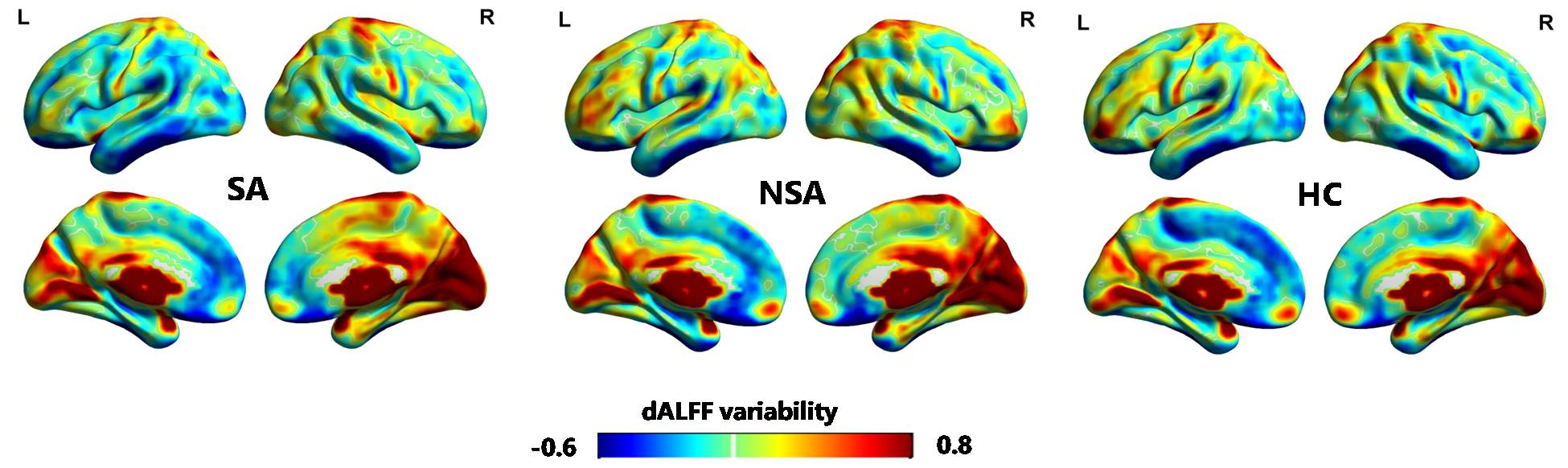


Figure S1. Spatial distribution of dALFF variability in the MDD-SA, MDD-NSA, and HC groups. Brain regions with high temporal variability were mainly located in the occipital cortices and postmedial, lateral frontal, parietal, limbic and sensorimotor cortices. SA, prior suicide attempt; NSA, no prior suicide attempt; HC, healthy control.


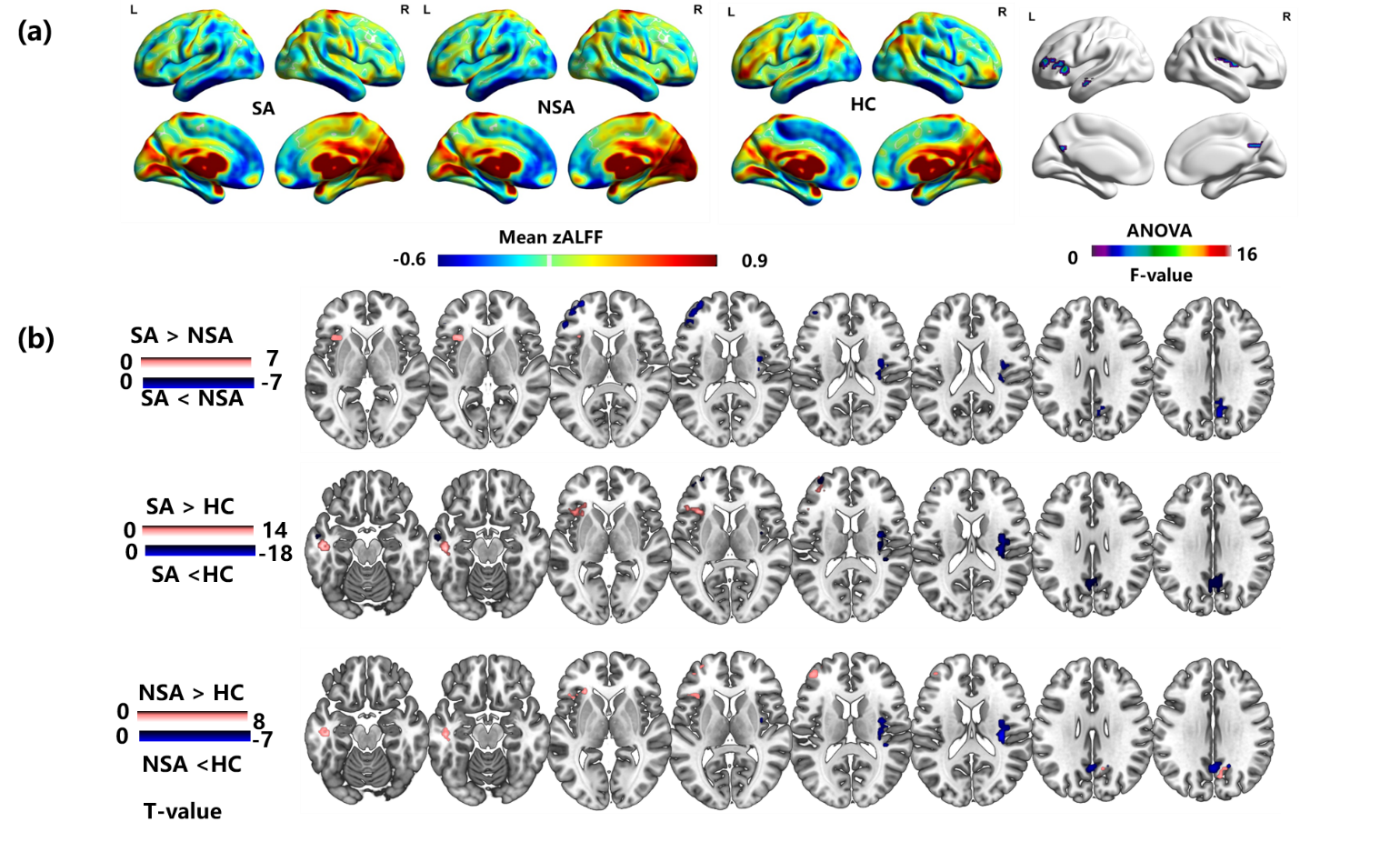


Figure S2. Spatial distribution pattern of sALFF and between-group differences. (a) Spatial distribution of mean sALFF in the MDD-SA, MDD-NSA, and HC groups and ANOVA results among the three groups. (b) Post hoc results among the MDD-SA, MDD-NSA and HC groups. The spatial distribution pattern and between-group differences of sALFF maps were highly consistent with that of the dALFF variability. SA, prior suicide attempt; NSA, no prior suicide attempt; HC, healthy control; sALFF, static amplitude of low-frequency fluctuations.


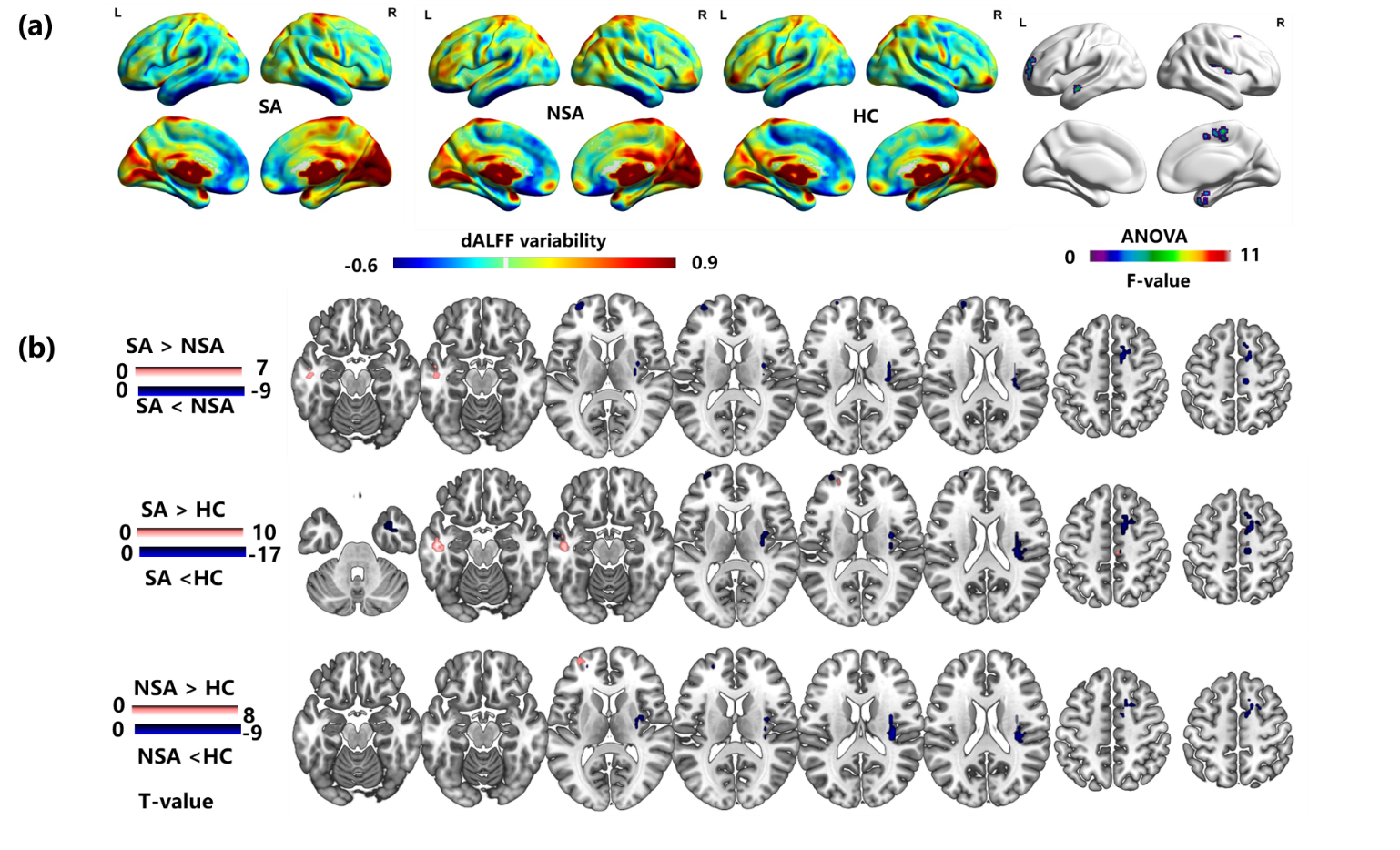


Figure S3. Results calculated with widow size of 40 TRs. (a) Spatial distribution of dALFF variability in the MDD-SA, MDD-NSA, and HC groups and ANOVA results among the three groups. (b) Post hoc results among the MDD-SA, MDD-NSA and HC groups. Results calculated with widow size of 40 TRs were highly similar to our main findings. SA, prior suicide attempt; NSA, no prior suicide attempt; HC, healthy control; dALFF, dynamic amplitude of low-frequency fluctuations.


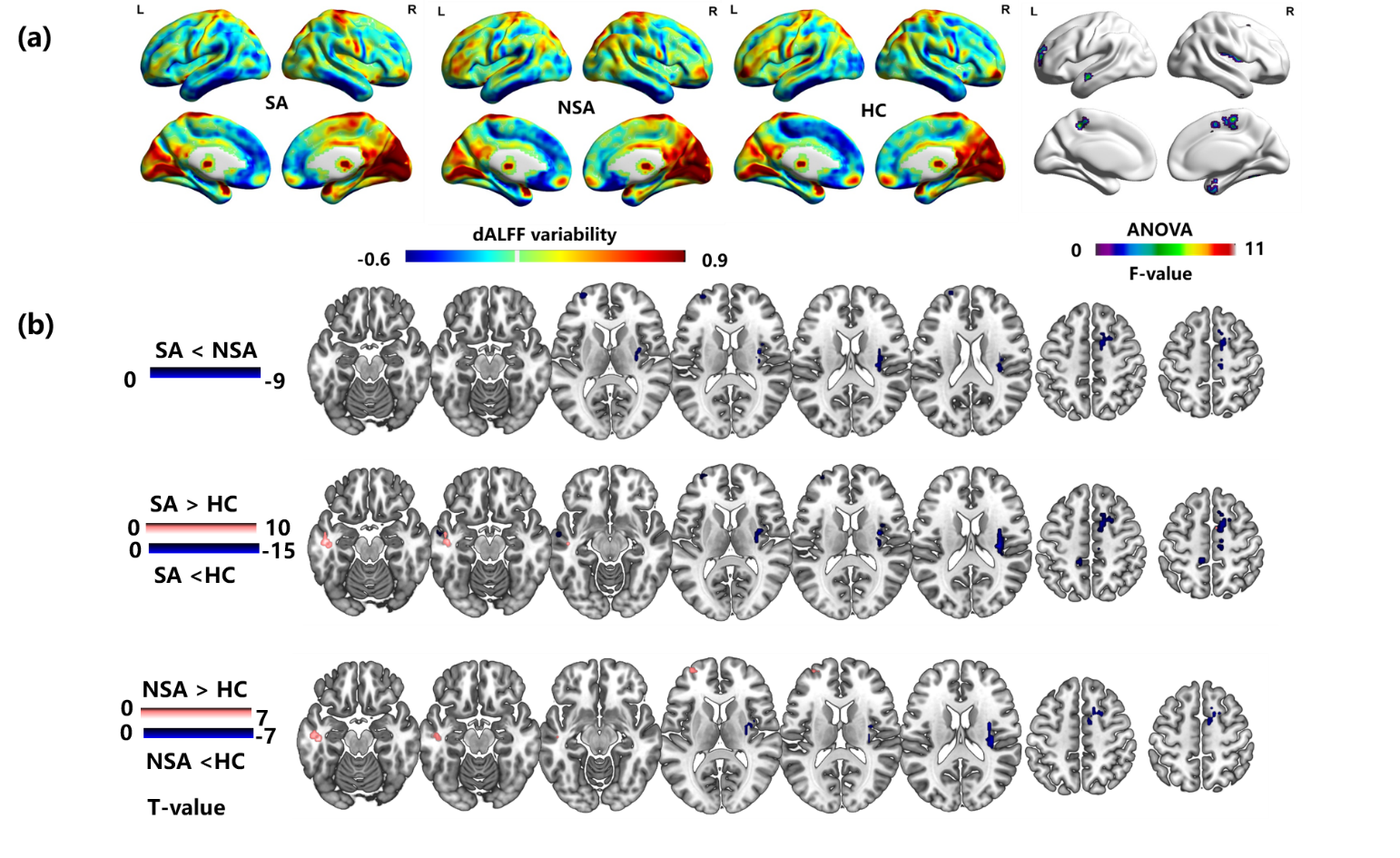


Figure S4. Results calculated with widow size of 50 TRs. (a) Spatial distribution of dALFF variability in the MDD-SA, MDD-NSA, and HC groups and ANOVA results among the three groups. (b) Post hoc results among the MDD-SA, MDD-NSA and HC groups. Results calculated with widow size of 50 TRs were also largely consistent with our main findings. SA, prior suicide attempt; NSA, no prior suicide attempt; HC, healthy control; dALFF, dynamic amplitude of low-frequency fluctuations.


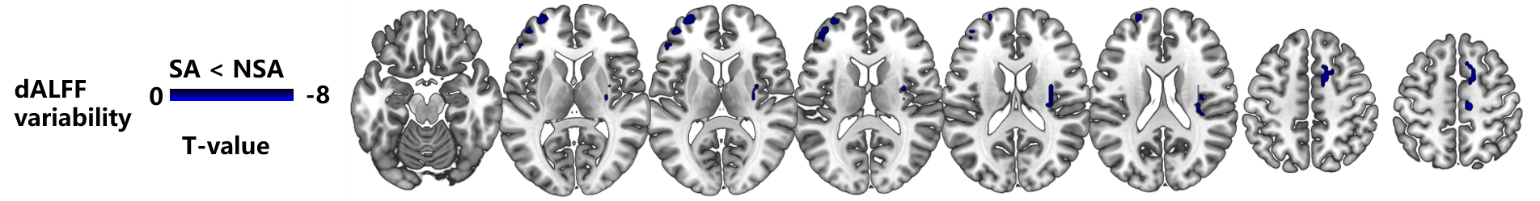


Figure S5. Post hoc analysis of dALFF variability between the MDD-SA and MDD-NSA groups with age at onset as an additional covariate. The result was highly similar to our main findings. SA, prior suicide attempt; NSA, no prior suicide attempt.


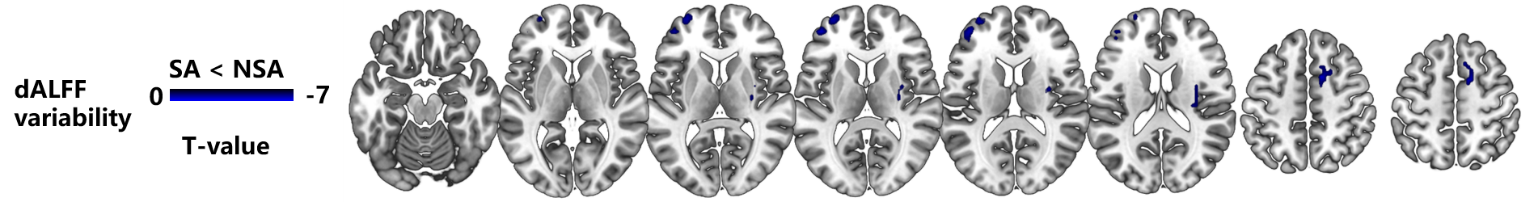


Figure S6. Post hoc analysis of dALFF variability between the MDD-SA and MDD-NSA groups with disease duration as an additional covariate. The result was highly similar to our main findings. SA, prior suicide attempt; NSA, no prior suicide attempt.
